# Essential Oil-Loaded Lipid Nanocarriers with Anti-Inflammatory Activity: Quality Control, Safety, and Efficacy

**DOI:** 10.3390/biomedicines14092027

**Published:** 2026-09-09

**Authors:** Pedro Paulo de Melo Ferreira, Ana Clara Santiago Bastos, Fernanda Nervo Raffin, Lígia Nunes de Morais Ribeiro

**Affiliations:** Department of Pharmacy, Faculty of Pharmacy, Federal University of Rio Grande do Norte, Natal 59012-570, RN, Brazil; ppmferreira700@gmail.com (P.P.d.M.F.); clara.santiago.101@ufrn.edu.br (A.C.S.B.); fernanda.raffin@ufrn.br (F.N.R.)

**Keywords:** nanotechnology, natural products, essential oils, lipid nanocarriers, anti-inflammatory activity

## Abstract

Inflammation is a complex biological response involved in the pathophysiology of several chronic diseases. Although conventional anti-inflammatory therapies are effective, their prolonged use can be associated with relevant adverse effects. Although non-steroidal anti-inflammatory drugs and glucocorticoids are widely used, these agents cause gastrointestinal, renal, cardiovascular, hepatic and metabolic adverse effects. Essential oils (EO) exhibit relevant anti-inflammatory potential. However, their pharmaceutical application is limited by volatility, hydrophobicity, physicochemical instability and potentially low bioavailability. This review evaluated EO-loaded lipid nanocarriers for anti-inflammatory applications, with emphasis on physicochemical quality attributes, stability, safety and efficacy. Literature searches were carried out through PubMed/MEDLINE, PubMed Central, ScienceDirect, SciELO, Virtual Health Library, and Google Scholar websites, based on works published between 2021 and 2026. The investigated systems included liposomes, solid lipid nanoparticles, nanostructured lipid carriers and nanoemulsions. Different formulations were found regarding EO nature, nanocarrier type, administration route and biological models used, predominantly involving preclinical assays. Nanoencapsulation was frequently associated with suitable physicochemical properties and release profiles, but direct comparisons with the corresponding free EO and control nanocarriers were not consistently performed. Safety findings were mainly based on cytotoxicity, local tolerability and short-term assays, with limited repeated-dose and long-term toxicological data. Several formulations showed anti-inflammatory activity in acute, subacute, or chronic experimental models. In selected works using the same experimental conditions, some formulations produced responses of similar magnitude to hydrocortisone, diclofenac, or other anti-inflammatory drugs, but these results cannot be directly correlated with therapeutic equivalence. The reported mechanisms involved modulation of the NF-κB, MAPK and Keap1/Nrf2/HO-1 signaling pathways. Overall, lipid-based nanocarriers represent platforms for investigating the stability, delivery and biological performance of EO. However, further standardization and controlled assays are required to determine their therapeutic and translational potential.

## 1. Introduction

Inflammation is an essential biological response for the defense of the organism. It acts in tissue repair, triggered by infectious, chemical, or physical stimuli capable of activating immune cells and signaling pathways involved in the production of cytokines, chemokines, eicosanoids and reactive species [1]. Although it represents a physiological mechanism indispensable for the maintenance of homeostasis, when persistent or dysregulated, the inflammatory response can contribute to the pathophysiology of different chronic diseases, favoring cellular damage, metabolic dysfunction and tissue remodeling [2]. In this context, it has been highlighted that the nuclear factor kappa B pathway, known as NF-κB, plays a central role in regulating the expression of genes associated with inflammation, immune response, proliferation and cell survival. Its abnormal activation is related to the maintenance of chronic inflammatory processes and the development of pathological alterations [3].

Despite the widespread use of nonsteroidal anti-inflammatory drugs (NSAIDs) and corticosteroids in clinical practice, these drugs present important limitations, especially when used for prolonged periods [4]. Panchal and Sabrina (2023) discussed that NSAIDs, although effective in controlling pain and inflammation, are currently associated with gastrointestinal, renal and cardiovascular adverse effects [5]. Moreover, Peinado-Acevedo et al. emphasized that glucocorticoids, despite being fundamental in modulating the inflammatory response, require caution due to the adverse effects resulting from excessive, prolonged, or inappropriate use [6]. Thus, the search for more efficient and selective delivery systems becomes relevant, aiming to reduce side effects, improve the bioavailability of bioactive compounds and enhance their action at the target site [7]. Table 1 summarizes the main NSAIDs currently employed in clinical practice, classified by their cyclooxygenase (COX) selectivity profile, oral dosage, prescription and most relevant side effects.

Essential oils (EO) are complex mixtures of volatile compounds produced by the secondary metabolism of aromatic plants. They mostly consist of terpenes, terpenoids, phenylpropanoids and other aromatic compounds [8]. These metabolites are responsible for organoleptic properties, as well as for biological activities, including antimicrobial, antioxidant, analgesic, antineoplastic and anti-inflammatory effects [9,10].

Cimino et al. (2021) highlighted that EO have broad biomedical potential, but their clinical application is limited by intrinsic physicochemical limitations, such as volatility, hydrophobicity, physicochemical instability, photosensitivity, hydrolysis, oxidative degradation and low absorption [11]. Although EOs represent promising sources of anti-inflammatory bioactive for the development of therapeutic candidates [9], several challenges remain to be overcome to enable their clinical use on a large scale [12].

**Figure 1:**
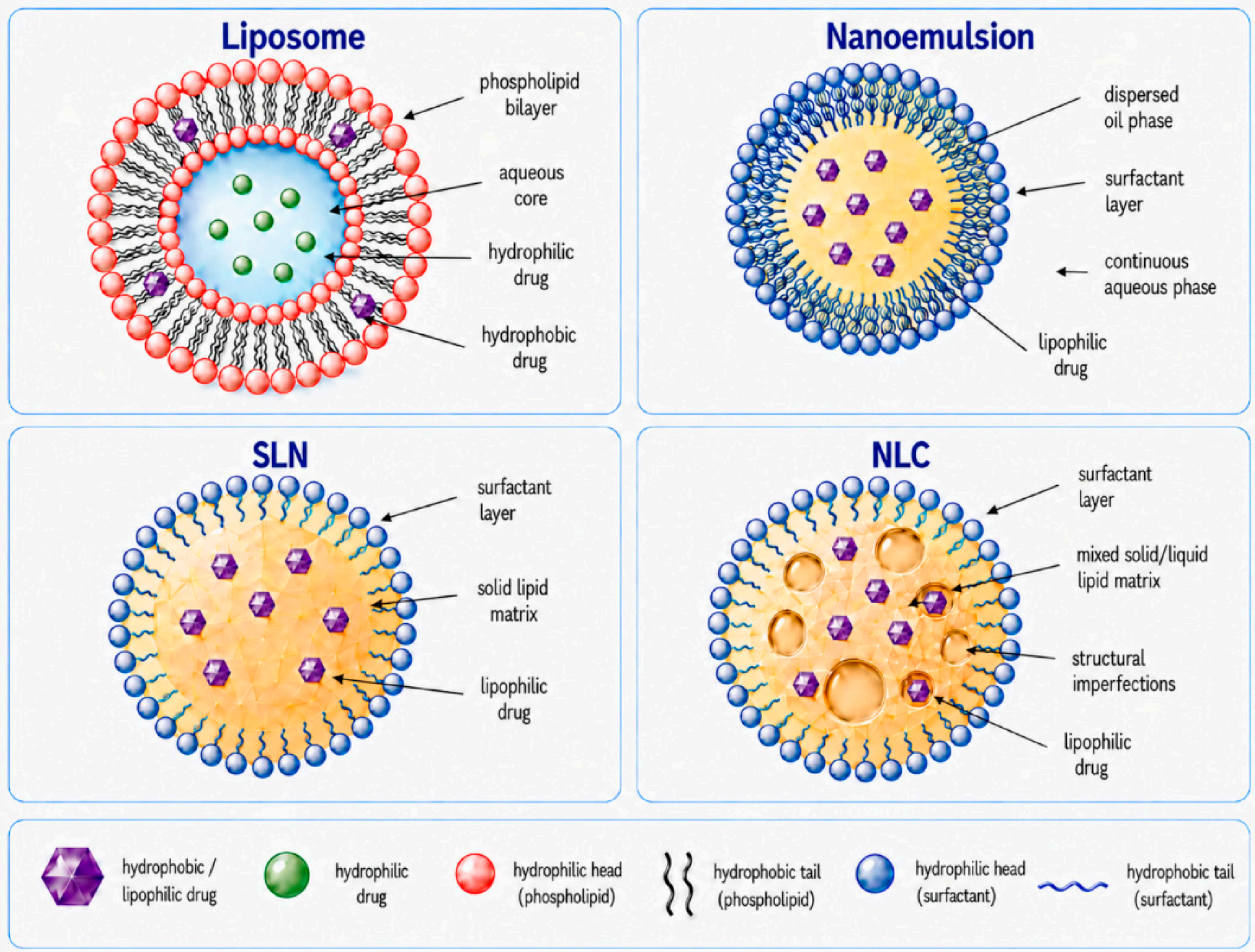
Schematic representation of the essential oil-based lipid nanoparticles: liposomes, nanoemulsions (NE), solid lipid nanoparticles (SLN) and nanostructured lipid carriers (NLC).

Nanostructured delivery systems have been widely investigated for the transport of natural compounds, such as terpenes, polyphenols, flavonoids and EO [13]. In agreement, Liu et al. (2024) demonstrated that nanotechnological approaches favored the bioavailability of poorly soluble drugs, especially through lipid, polymer and hybrid nanoplatforms [14]. Kamble et al. (2025) pointed out that the nanoencapsulation of bioactive compounds can increase stability, promote sustained release and improve bioavailability in biological systems [15]. Among the available nanocarriers, lipid-based systems such as liposomes, solid lipid nanoparticles (SLN), nanostructured lipid carriers (NLC) and nanoemulsions (NE) stand out for their biocompatibility, versatility and suitability for the encapsulation of hydrophobic molecules, supporting their potential as platforms for the delivery of anti-inflammatory compounds. These lipid-based nanocarriers differ in their structural organization and composition, as schematically illustrated in Figure 1.

Liposomes are spherical vesicles composed of one or more phospholipid bilayers surrounding an aqueous core, enabling the incorporation of hydrophilic, hydrophobic and amphiphilic compounds. Although they exhibit high biocompatibility and low immunogenicity, they can be limited by physicochemical instability, relatively high production costs, and the need for cold storage. On the other hand, SLN consist of a surfactant-stabilized matrix of solid lipids, providing protection and sustained release of hydrophobic compounds. However, their highly ordered crystalline structure can restrict drug loading and cause active-ingredient expulsion during storage. NLC combine solid and liquid lipids to generate a less ordered matrix, increasing loading capacity and improving the retention of hydrophobic compounds compared with SLN. However, their stability and performance remain dependent on lipid composition and formulation conditions. NE are nanoscale oil–water dispersions, offering simple and low-cost preparation and exhibiting narrow size distribution. Nevertheless, they can show some toxicity, due to the high concentrations of the surfactant and co-surfactant phases in their composition [8].

This review aimed to critically analyze the available reports regarding lipid nanocarriers containing EO with anti-inflammatory activity. Previous literature reviews have focused on the discussion of anti-inflammatory EO and the EO nanoencapsulation technologies separately. Here, we integrated nanocarriers and EO characterization, physicochemical stability, safety and anti-inflammatory activity data as an indissociable supramolecular entity. Particular attention was directed to the biological assays, including comparisons with free EO and control formulations. In addition, the main technological and translational limitations associated with the development of these systems were discussed.

## 2. Methods

This work included published works that addressed the development, physicochemical characterization, safety and anti-inflammatory efficacy of lipid-based nanocarriers loading EO. SLN, NLC, liposomes, and NE were included. The analyses focused on the technological and physicochemical properties of the formulations, as well as on evidence of their safety and anti-inflammatory activity obtained from in vitro and in vivo experiments.

The literature search was conducted using the PubMed/MEDLINE, PubMed Central, ScienceDirect, SciELO, Virtual Health Library and Google Scholar databases. English-language search terms were combined using the Boolean operators AND and OR. The main terms included “essential oils”, “lipid nanoparticles”, “nanostructured lipid carriers”, “solid lipid nanoparticles”, “nanoemulsions”, “liposomes”, “anti-inflammatory activity”, “inflammation”, “cytotoxicity”, “physicochemical stability” and “quality control”. The search strategies were adapted to the specific characteristics of each source. Representative search combinations included: “Essential oils” AND “nanostructured lipid carriers” AND “anti-inflammatory activity”; “essential oils” AND “solid lipid nanoparticles” AND “inflammation”; “essential oils” AND “nanoemulsions” AND “anti-inflammatory activity”; “essential oils” AND “liposomes” AND “cytotoxicity”. Articles published between 2021 and 2026 were considered.

## 3. Overview

The works included in this review were organized according to the type of lipid nanocarrier and their main thematic contribution. Formulations based on liposomes, SLN, NLC and NE containing EO with anti-inflammatory activity were identified, while additional publications were used only for conceptual contextualization. Table 2 consolidated the overview with comparative information of the selected publications regarding the formulation properties, experimental conditions, controls, anti-inflammatory findings and safety assessments. Comparisons between nanoformulations and free EO were reported when the data were available.

## 4. Inflammatory Process and Conventional Drug Therapies

Inflammation is the stereotyped and evolutionarily conserved response of vascularized tissues to harmful stimuli, including infection, mechanical injury, chemical irritation and metabolic stress. Medzhitov (2021) discussed inflammation not as a single symptom, but as a spectrum of responses ranging from canonical host defense to para-inflammation and chronic low-grade inflammation, with distinct adaptive logic and pathological consequences [1]. Acute inflammation, classically characterized by the cardinal signs (rubor, heat, tumor, pain and loss of function), is short-lived and self-resolving. Chronic inflammation is sustained, often silent and increasingly recognized as a central driver of human disease. Immune-mediated inflammation results from dysregulated or inappropriate activation of the immune system. Metabolic inflammation is a sustained inflammatory state, driven by metabolic dysfunction, nutrient excess, or adipose-tissue stress. Furman et al. (2019) attributed a substantial fraction of the global non-communicable disease burden, such as cardiovascular, neurodegenerative, metabolic, oncological and autoimmune disorders, to chronic, systemic, low-grade inflammation [2].

At the cellular and molecular level, the inflammatory cascade is orchestrated by a coordinated network of mediators. Pro-inflammatory cytokines, such as tumor necrosis factor alpha (TNF-α), interleukin-1 beta (IL-1β) and interleukin-6 (IL-6) initiate and amplify the response. Inducible enzymes such as cyclooxygenase-2 (COX-2) and inducible nitric oxide synthase (iNOS) generate prostaglandins and nitric oxide that perpetuate vasodilation, hyperalgesia and tissue remodeling [1,3]. The nuclear factor kappa B (NF-kappaB) pathway lies at the center of this network: upon activation by pattern-recognition receptors, cytokine receptors, or stress signals, NF-kappaB translocates to the nucleus and induces transcription of dozens of pro-inflammatory genes. Mao, Zhao and Sun (2025) mapped the role of NF-kappaB in chronic inflammation and cancer, underlining how its persistent activation links inflammation to malignant transformation [3]. Targeting one or several nodes of this network: cytokines, COX-2, iNOS and NF-kappaB, remains the conceptual basis of anti-inflammatory pharmacotherapy.

Conventional pharmacological therapy relies on the oral administration of two classes of drugs: non-steroidal anti-inflammatory drugs (NSAIDs) and glucocorticoids. NSAIDs act primarily through inhibition of the COX-1 and/or COX-2 isoforms, reducing prostaglandin synthesis. Despite well-established efficacy against pain and inflammation, NSAID use is constrained by a clinically relevant side-effect profile. Bindu, Mazumder and Bandyopadhyay (2020) provided a thorough mechanistic account of NSAID-induced organ damage, with emphasis on the gastrointestinal tract, kidneys and liver, linking these effects to the depletion of cytoprotective prostaglandins [4].

Subsequent literature has refined the NSAID risk profile. Domper Arnal, Hijos-Mallada and Lanas (2022) reviewed the gastrointestinal and cardiovascular adverse events associated with both non-selective and COX-2-selective NSAIDs [16]. They highlighted the persistence of upper gastrointestinal bleeding risk and the dose-dependent cardiovascular signal even with coxibs. More recently, Ahsan and Sahu (2025) consolidated evidence on NSAIDs and cardiovascular events, reporting risk gradients that vary by molecule, dose, and patient comorbidity [17]. They recommended stratified prescribing in high-risk populations [17]. These adverse profiles are particularly problematic in chronic inflammatory conditions, where long-term use is the rule rather than the exception.

Glucocorticoids are widely used anti-inflammatory and immunosuppressive agents that act through genomic and non-genomic mechanisms, including the repression of NF-κB and AP-1 activity and the induction of anti-inflammatory genes. Although they play an important role in the treatment of inflammatory and immune-mediated disorders, systemic glucocorticoid therapy is associated with substantial adverse effects, including hyperglycemia, osteoporosis, weight gain, hypothalamic–pituitary–adrenal axis suppression, increased susceptibility to infections and neuropsychiatric effects [18].

Peinado-Acevedo et al. (2024) concluded that glucocorticoid toxicity is dose- and duration-dependent and recommended keeping long-term prednisolone-equivalent doses below 7.5 mg/day, since chronic exposure above this threshold is consistently associated with irreversible organ damage and increased mortality in autoimmune diseases [6]. Bergstra et al. (2023) in the systematic literature review analyzed the European Alliance of Associations for Rheumatology (2022) recommendations for rheumatoid arthritis [19]. It was confirmed that long-term glucocorticoid use significantly increases the risk of osteoporotic fractures, serious infections, diabetes mellitus and mortality, with safety risks rising in parallel with dose and duration [19].

It is worth mentioning that the route of administration strongly influences the therapeutic success of anti-inflammatory drugs by determining systemic exposure, tissue distribution, onset of action and the intensity of adverse effects. Oral administration is generally effective for acute and chronic inflammatory conditions, as it is convenient and facilitates patient compliance. However, therapeutic failure or dose limitation can result from variable gastrointestinal absorption, first-pass metabolism, poor adherence and gastrointestinal, renal, or cardiovascular toxicity associated with prolonged use [20]. Topical and transdermal administration can be successful for localized and superficial inflammatory disorders, as it increases drug deposition at the site of inflammation, while reducing systemic exposure. Nevertheless, efficacy should be inadequate when the target tissue is deep or when the drug has poor skin permeation, as the stratum corneum acts as a substantial barrier [21]. Parenteral administration, particularly intravenous or intramuscular, provides rapid and relatively predictable systemic exposure. Therefore, it is advantageous in acute, severe, or postoperative inflammation and when oral administration is not feasible. Conversely, its invasive nature, limited suitability for long-term treatment, injection-related complications and potential for systemic toxicity restrict its therapeutic use [22].

Together, the gastrointestinal, cardiovascular and renal risks of NSAIDs and the metabolic, endocrine and infectious risks of glucocorticoids define the therapeutic gap that motivates the search for safer alternatives [4,6]. In this context, naturally derived anti-inflammatory compounds have attracted attention as therapeutic agents or adjuvants. Among them, EO have been investigated for their ability to modulate the same molecular sites targeted by conventional drugs (NF-kappaB, COX-2, iNOS, and cytokines), often with a multitarget, rather than a single-target, action [9,23,24].

## 5. Essential Oils as Bioactive Compounds

EO are concentrated, volatile and complex mixtures obtained primarily from aromatic plants by hydrodistillation, steam distillation, cold pressing (for citrus peels), or, more recently, supercritical fluid extraction. De Sousa et al. (2023) emphasized that despite their simple botanical origin, EO are chemically intricate matrices containing dozens to hundreds of bioactive constituents, whose relative proportions define each oil chemotype [9]. Ben Miri (2025) further consolidated this perspective in a comprehensive review of EO chemistry and bioactivity, framing the field around the dialogue between phytochemical complexity and pharmacological outcome [25]. Chemically, EO are composed of terpenes/terpenoids and phenylpropanoids. Monoterpenes (C10), such as limonene, α-pinene, β-pinene, linalool, thymol, carvacrol, 1,8-cineole, and menthol and sesquiterpenes (C15), such as β-caryophyllene, α-humulene and farnesene, arise mainly through the mevalonate (MVA) and methylerythritol phosphate (MEP) pathways. Phenylpropanoids such as eugenol, cinnamaldehyde and dillapiole originate from the shikimate pathway via L-phenylalanine. In addition, EO can exert a dual role in the system, acting as therapeutic agents and as natural penetration enhancers in topical and transdermal formulations [13].

The pharmacological application of EO is broad. Antimicrobial activity has historically been the most studied attribute, but antioxidant, antitumoral, anxiolytic, antinociceptive, and anti-inflammatory effects are equally well-documented [10]. For the anti-inflammatory phenotype, some converging lines of evidence are now standard. Several EO and isolated terpenes inhibit pro-inflammatory transcription factors, particularly NF-κB, with downstream reduction in TNF-α, IL-6, and iNOS expression. Yang et al. (2023) [23] reviewed the anti-inflammatory effects of citrus EO (limonene-rich), reporting consistent suppression of NF-κB signaling and cytokine release in LPS-stimulated RAW 264.7 macrophages. Phenolic monoterpenes, such as thymol and carvacrol, act as direct antioxidants, mitigating reactive oxygen species (ROS) that drive inflammasome activation [23]. Gago et al. (2025) reviewed the anti-inflammatory activity of thymol and thymol-rich EO, integrating in vitro, in vivo and in silico evidence, describing inhibition of COX-2 and iNOS as recurrent mechanisms [24]. Several EO exhibit antinociceptive properties that complement their anti-inflammatory action, an advantage in conditions where pain and inflammation occur together [9].

Free EO face well-known limitations that constrain their pharmaceutical use. Their constituents are highly volatile and chemically labile, prone to oxidation, polymerization, and isomerization on exposure to light, heat and oxygen. Their hydrophobicity translates into low aqueous solubility and low absorption, and can be irritating to skin and mucosa [11]. Albuquerque et al. (2022) summarized the biotechnological applications of nanoencapsulated EO and explicitly their disadvantages, such as volatility, oxidative instability, low bioavailability, organoleptic drawbacks and dose-dependent irritancy, as the main reasons why their nanoencapsulation is necessary [26]. Kaspute et al. (2025) reinforced this argument by noting that, even when the molecular pharmacology is favorable, the macroscopic behavior of free EO (rapid evaporation, oil–water phase separation, sensorial issues) can prevent its clinical use [13].

## 6. Pharmaceutical Nanotechnology

Pharmaceutical nanotechnology refers to the rational design, preparation, characterization and biomedical application of structures with at least one dimension in the nanometric range. In this sense, nanocolloidal dispersions are widely studied for multipurpose applications. They have predictable size and surface charge, showing sustained release profiles of encapsulated bioactive. Therefore, a nanocarrier is conceived as a functional entity that modulates absorption, intracellular uptake, biodistribution and excretion [7,27].

The pharmaceutical relevance of the carriers at the nanometric scale arises from a set of physicochemical properties that differ from the micro- and macroscale. The miniaturization process increases the surface-area-to-volume ratio by several orders of magnitude, which enhances the external interfacial area to interact with biological barriers. It also contributes to colloidal surface phenomena, such as adsorption, charge density, or adhesion [14]. These properties are particularly relevant for the nanoencapsulation of hydrophobic, natural-based actives, such as EO, which also combine several therapeutic properties with intrinsic physicochemical instability [9,28,29].

## 7. Nanostructured Delivery Systems

**Figure 2:**
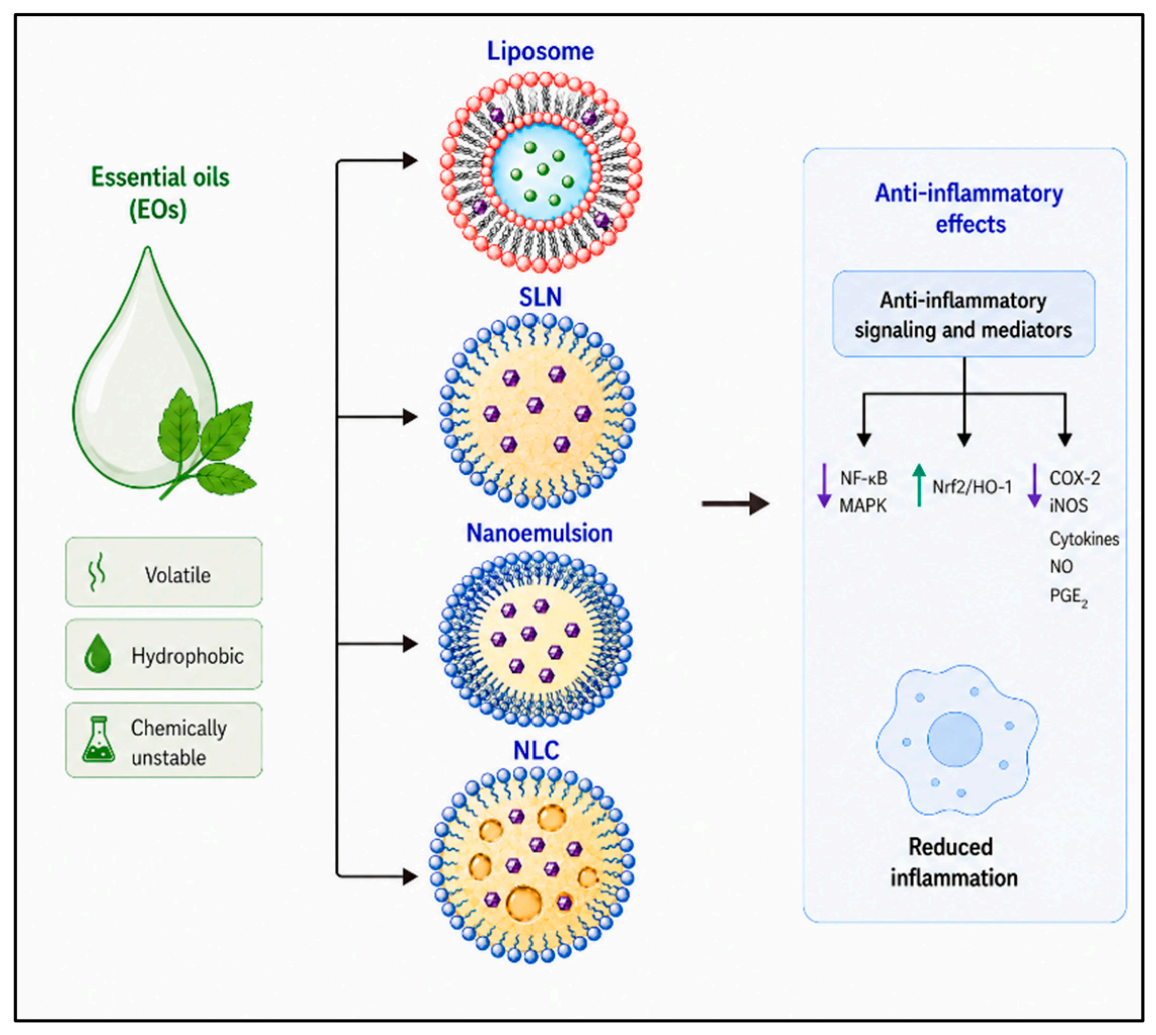
Schematic representation of the mechanisms of action of the anti-inflammatory essential oil-based lipid nanoparticles.

Lipid-based nanocarriers occupy a particularly favorable position for the encapsulation of EO, given the chemical compatibility between the lipid nature of the carrier and EO [7]. The following section displays the main lipid nanocarriers that load EO with anti-inflammatory activity, as follows: liposomes, SLN, NLC and NE. Figure 2 shows the main mechanisms of action related to EO-based lipid nanoparticles with anti-inflammatory activity.

### 7.1. Liposomes

Mirković et al. (2025) developed conventional liposomes based on Phosal 40 IP, a phosphatidylcholine matrix (40% non-GMO unsaturated phosphatidylcholine in organic sunflower seed oil) to entrap the EO obtained by Clevenger from green cones of *Pinus halepensis* [30]. The formulation (EO at 1% *w*/*w*) revealed a mean particle size of 197.4 ± 3.81 nm, polydispersity index of 0.142 ± 0.016, zeta potential of −35.61 ± 1.45 mV and encapsulation efficiency of 97.35 ± 0.82%. Such structural parameters were preserved over one year of storage at room temperature. Safety was inferred through an in vivo tolerability model in male Wistar rats, without observable signs of cutaneous irritation. Anti-inflammatory efficacy was evaluated in a carrageenan-induced paw edema model in rats. Topical application of 0.3 g of liposomes produced edema inhibition of 51.83 ± 4.12% after 3 h and 69.23 ± 5.34% after 4 h. These results were statistically equivalent to topical administration of 1% hydrocortisone ointment acting in the late phase of inflammation [30].

Other work developed ultradeformable nanocarriers called transfersomes. It is a liposomal variety composed of Phospholipon 90G^®^ (88 mg) and sodium cholate as an edge activator (12 mg) diluted in a hydroalcoholic medium (water:ethanol 93:7 *v*/*v*), co-loaded with bergamot EO (BEO) and ammonium glycyrrhizinate (AG) from *Glycyrrhiza glabra* L. Vesicles were prepared by thin-layer evaporation followed by the extrusion method. Physicochemical characterization revealed a mean particle size of 119.0 ± 1.0 nm to 129.9 ± 1.0 nm, a polydispersity index of 0.120 ± 0.024, zeta potential of −43.2 ± 1.6 mV, and encapsulation efficiency of 77.8 ± 1.0% for AG. Long-term physicochemical stability was also shown. Safety was assessed in 10 healthy human volunteers, with no statistically significant erythema index variation versus saline solution. Anti-inflammatory efficacy was evaluated in methyl nicotinate-induced cutaneous erythema in the same volunteers, where the BEO + AG co-loaded vesicle produced a significant reduction of the lesion index compared with the AG-only vesicle (*p* < 0.001), caused by TNF-α, IL-6, COX-2, iNOS, and PGE2 modulation [31].

Hou et al. (2026) developed PEG-stabilized bilayer nanodisks, which are another variety of liposomes [32]. They were composed of DSPC, cholesterol, and DSPE-MPEG2000 loaded with garlic oil (*Allium sativum* L.; CAS 8008-99-9), an organosulfur species that includes diallyl sulphide, diallyl disulphide, and diallyl trisulphide as the principal Nrf2-binding constituent. The formulation was prepared by thin-film dispersion followed by sonication. Physicochemical characterization revealed a mean particle size of 148.0 ± 3.0 nm, polydispersity index of 0.150 ± 0.02, and zeta potential of −0.2 ± 0.1 mV (consistent with PEG-mediated steric shielding). Encapsulation efficiency of 55.26 ± 0.04%, with colloidal stability confirmed in water, HEPES, and phosphate buffer (pH 5.8) at 4 °C, 25 °C, and 40 °C. Safety was not analyzed in this work. Anti-inflammatory efficacy was evaluated in LPS-induced acute lung injury in male ICR mice (LPS 15 mg/kg intraperitoneally; nanodisks 50 mg/kg by tail-vein injection). The formulation significantly reduced bronchoalveolar lavage protein, lung wet/dry ratio, TNF-α and IL-6, increased IL-4, IL-10, SOD, CAT and total antioxidant capacity, decreasing malondialdehyde and nitric oxide, with mechanistic confirmation by Western blot of NF-κB p65 inhibition coupled with Keap1–Nrf2/HO-1/NQO1 activation [32].

Another work, conducted by Ding et al. (2023), described the development of flexible liposomes co-loaded with total glucosides of peony (TGP; root extract of Paeonia lactiflora Pallas, paeoniflorin being the principal active) and EO from the twigs of *Cinnamomum cassia*, as a transdermal anti-inflammatory formulation [33]. The incorporation of *C. cassia* EO did not significantly alter the structural properties of TGP-loaded flexible liposomes. Anti-inflammatory efficacy was evaluated in an arthritis rat model, in which the transdermal application of the EO-based flexible liposomes significantly reduced the paw edema and serum inflammatory cytokine levels even at lower doses. The in vitro permeation assays demonstrated 4.6, 1.8, and 1.5-fold higher TGP skin flux versus TGP solution, the TGP + physical mixture of EO and plain TGP-loaded flexible liposomes, respectively [33].

### 7.2. Solid Lipid Nanoparticles

Kotb et al. (2023) developed SLN composed of Compritol^®^ 888 ATO (glyceryl behenate, 1% *w*/*w*) and stabilized with Tween^®^ 80 (4% *w*/*w*), loading *Boswellia papyrifera* EO [34]. Physicochemical characterization revealed nanosized spherical particles, sustained in vitro release over 8 h, high entrapment efficiency, and stability at 5 °C and 25 °C and 60% RH for 3 months. Safety was assessed in vivo on a rat skin model. It showed healthy epidermal/dermal architecture and reduced inflammatory infiltrate in the formulation treatment group. Anti-inflammatory efficacy was evaluated in male Wistar rats exposed to UVB at 40–80 mJ/cm^2^ for 10 consecutive days. They were topically treated daily. The formulation significantly reduced cutaneous IL-6 and NF-κB p65 by ELISA, downregulated phospho-ERK, phospho-JNK, and phospho-p38 MAPK and the PI3K/AKT axis by Western blot, upregulated TGF-β1 by qPCR, and restored superoxide dismutase and catalase activities [34].

### 7.3. Nanostructured Lipid Carriers

Mohemmad et al. (2024) developed NLC composed of cocoa butter as the solid lipid and extra-virgin olive oil as the liquid lipid, stabilized by Tween 80, loading *Elettaria cardamomum* EO [35]. Physicochemical characterization indicated a mean particle size of 150.13 ± 15.25 nm, PDI of 0.449 ± 0.45, and %EE of 91.95% ± 0.85%, with physicochemical stability maintained over 28 days. Safety was assessed by histological inspection of the liver, pancreas and kidney in male Wistar rats, in which no relevant alterations were detected. Anti-inflammatory efficacy was evaluated in an ethanol-induced gastric ulcer model in male Wistar rats (300 and 600 mg/kg orally), compared with omeprazole at 20 mg/kg. NLC significantly reduced IL-6 and TNF-α, increased IL-10 and decreased MDA, with a concomitant reduction in gastric ulcer area and gastrin levels [35]. Ahmed et al. (2024) evaluated the same NLC formulation described above, which was developed to act simultaneously as a hypoglycemic and anti-inflammatory agent [36]. The formulation was well tolerated in male Wistar rats during four consecutive weeks of administration, with no relevant hepatic, pancreatic, or renal alterations. Its effects were assessed in rats with diabetes induced by a high-sugar/high-fat diet combined with streptozotocin administration (STZ, 50 mg/kg), using empagliflozin as the positive control. After 28 days, the NLC formulation reduced fasting blood glucose from approximately 450 mg/dL in the diabetic control group to 250 mg/dL, corresponding to a 44.4% reduction. During the same period, the empagliflozin-treated group showed a blood glucose level of approximately 300 mg/dL. The formulation also reduced body weight and the levels of the pro-inflammatory biomarkers TNF-α and IL-6, while contributing to the preservation of pancreatic islet histology [36].

Shakeel et al. (2021) developed another NLC containing clove essential oil for the topical treatment of chronic arthritic inflammation [37]. Physicochemical characterization revealed a mean particle size of 120.0 ± 1.42 nm, a polydispersity index of 0.148 ± 0.012, a zeta potential of −27.56 ± 1.0 mV and an encapsulation efficiency of 84.53 ± 1.15%. The NLC formulation remained physicochemically stable for 90 days under storage conditions of 25 °C and 40 °C/65% relative humidity. In vitro anti-inflammatory activity was demonstrated by 82.73 ± 1.63% inhibition of protein denaturation, compared with 86.91 ± 1.34% for Voltaren^®^. Local dermal tolerability was supported by the absence of observable signs of skin irritation in rats. Anti-inflammatory efficacy was evaluated in a Freund’s complete adjuvant-induced arthritis model in male Wistar rats. The animals received the topical clove essential oil-loaded NLC formulation for 21 days, using 1% diclofenac sodium gel as the positive control. The nanoformulation significantly reduced paw edema volume and decreased serum levels of lysosomal enzymes, TNF-α, IL-1β, and IL-6, with effects reported as comparable to those of diclofenac [37]. Carneiro and coworkers (2022) developed a hydrogel-thickened NLC loaded with dillapiole-rich *Piper aduncum* EO [38]. Structural analyses revealed spherical particles with a homogeneous size distribution of 131.3 ± 1.2 nm, PDI of 0.231 ± 0.003 and zeta potential around −42.1 ± 2.0 mV. Monitoring of physicochemical stability confirmed a shelf life of 90 days under storage at 4 °C and 25 °C. Nanotoxicity assays on a chicken embryo model showed that the formulation, applied topically to the chorioallantoic membrane, caused low irritation. In addition, in vitro permeation through a porcine skin model demonstrated that the NLC was delivered to the dermis, the biological layer of interest for anti-inflammatory action [38].

### 7.4. Nanoemulsions

Abdelhameed et al. (2023) developed an NE loaded with *Eugenia supra-axillaris* EO [28]. Structural organization was revealed, with droplet size of 88.0 ± 4.2 nm and neutral zeta potential of −1.84 ± 0.12 mV, polydispersity index and %EE were not available. Safety was assessed by acute oral toxicity, with no mortality or toxic signs up to 2000 mg/kg (LD50 > 2000 mg/kg), confirming a non-hemolytic profile. Anti-inflammatory efficacy was evaluated in a carrageenan-induced rat paw edema model, with oral dosing at 50 and 100 mg/kg. NE (100 mg/kg) achieved 53.61 ± 3.15% edema inhibition after 24 h, comparable to celecoxib and superior to short-acting diclofenac, alongside analgesic and antipyretic activity [28]. Another formulation containing *Pelargonium graveolens* EO was described by Gavzan et al. as an NE [39]. The system exhibited a mean droplet diameter of approximately 553.1 ± 3.1 nm, a polydispersity index of 0.113 ± 0.007, a positive zeta potential of +28.4 ± 1.2 mV and a spherical morphology. Despite the narrow size distribution, the reported mean droplet diameter is more consistent with a macroemulsion than with a NE [39]. Safety was confirmed by a non-hemolytic result prior to in vivo testing. Anti-inflammatory efficacy was evaluated in formalin- and carrageenan-induced paw edema models in mice. The NE (50 and 100 mg/kg) significantly inhibited edema during the 1–5 h carrageenan phase and over 24 h in the formalin model, whereas the free oil (10 mg/kg) was inactive. The formulation also produced anti-nociceptive effects in the hot-plate and acetic-acid writhing models, which were reversed by an opioid antagonist, suggesting involvement of the opioid pathway, an analgesic mechanism distinct from its anti-inflammatory action [39].

Gava and coworkers (2025) prepared a NE loaded with *Ocimum gratissimum* EO, with a droplet size of 112.0 ± 1.2 nm, PDI of 0.215 ± 0.009 and a slightly negative zeta potential (−5.0 ± 0.4 mV, indicating steric rather than electrostatic stabilization) [40]. Physicochemical stability was monitored up to 60 days at 4 °C. In the biocompatibility assays, the formulation showed low ocular irritability (HET-CAM and CAM-TBS models), no phototoxicity (NRU-3T3 assay) and no cytotoxicity to L929 (94.3 ± 2.5%), RAW 264.7 (92.1 ± 3.1%) or HaCaT cells (95.6 ± 1.8%). In vitro anti-inflammatory efficacy was evaluated in LPS-stimulated RAW 264.7 macrophages (1 µg/mL LPS): the NE reduced intracellular reactive oxygen species (superoxide and hydrogen peroxide) and inhibited TNF-α, IL-6 and IL-1 production at 2.5 and 5 µg/mL EO, whereas the free EO suppressed only TNF-α [40].

Anti-inflammatory nanoemulgels have also been described with success. A nanoemulgel containing *Melissa officinalis* EO was reported. Physicochemical characterization revealed a droplet size of 127.31 ± 2.84 nm, polydispersity index of 0.177, zeta potential of −25.0 ± 1.5 mV and %EE of 95.63 ± 0.47%, with spherical morphology by scanning electron microscopy. Anti-inflammatory efficacy was evaluated in a carrageenan-induced Wistar rat paw edema model, in comparison with diclofenac gel (2.5% *w*/*w*). At 30 min, the nanoemulgel reduced paw edema volume to 0.25 mL versus 0.32 mL for diclofenac [41]. Mohammadifar and coworkers (2021) developed a NE gel loaded with *Mentha piperita* and *Rosmarinus officinalis* EO [42]. Bioassays were conducted on acute dermal irritation model in New Zealand White rabbits, and acute dermal toxicity assay in Wistar rats. No adverse local reactions were observed over the 14-day period and no relevant changes in hematological or biochemical parameters. Anti-inflammatory efficacy was evaluated in a monosodium iodoacetate-induced knee osteoarthritis model in male rats over 14 days, with a diclofenac gel as control. In this model, NE gel significantly increased the paw-withdrawal threshold and reduced the withdrawal frequency (*p* < 0.01), increased the superoxide dismutase and glutathione peroxidase antioxidant enzymes, decreased malondialdehyde and improved the inflamed synovial membrane by histology. The anti-inflammatory effect of the NE gel was similar to or higher than that of diclofenac [42].

Yetukuri and Umashankar (2025) developed a nanoemulgel of *Kunzea ericoides* EO optimized by a Central Composite Design approach [43]. Structural data revealed a mean droplet size of 112.38 ± 1.45 nm, polydispersity index of 0.203 ± 0.012 and zeta potential of −29.0 ± 1.1 mV. Acute dermal irritation assay was carried out in healthy male Wistar rats. The nanoemulgel was confirmed to be a non-irritant form. Anti-inflammatory efficacy was evaluated in a carrageenan-induced paw edema model in rats, with 2.5% Dicloran^®^ gel as the control. The optimized nanoemulgel produced a statistically significant reduction in edema (*p* = 0.005 at 60 min), comparable to the diclofenac reference [43]. Esmaeili and coworkers (2022) developed NE-based nanogels loaded with *Cinnamomum zeylanicum* and *Syzygium aromaticum* EO [44]. In this work, topical tolerability was inferred from the absence of behavioral distress in the animals during testing. Anti-inflammatory efficacy was evaluated using a carrageenan-induced paw edema model, together with the hot-plate and formalin tests in male Wistar rats, covering both acute and chronic phases. The nanogels produced significant anti-inflammatory and anti-nociceptive activity, with the latter driven by the chronic phase of the formalin test, whereas no significant effect was observed in the hot-plate test. The cinnamon-based nanogel was more effective in the chronic phase [44]. Another work reported a NE encapsulating *Eucalyptus cladocalyx* EO. Physicochemical characterization displayed droplet size of approximately 40.0 ± 1.45 nm and polydisperse distribution, with PDI of 0.490 ± 0.03, followed under different storage conditions for 4 months. Anti-inflammatory efficacy was evaluated in lipopolysaccharide-stimulated RAW 264.7 macrophages, where both NE and free EO significantly inhibited the production of NO, IL-6 and TNF-α, indicating modulation of the NF-κB axis (*p* < 0.05) [45]. A topical NE cream incorporating 10% (*v*/*v*) *Syzygium aromaticum* (clove) EO was also reported. A mean droplet size of approximately 190 nm, polydispersity index of 0.08 and %EE of 94.54% indicated a narrow size distribution with high upload ability. Anti-inflammatory efficacy was evaluated in mice. Clove-oil-based NE cream produced significant anti-inflammatory activity together with antifungal effect (MIC 120 µL/mL) [46].

**Table 2:** Overview of published works of essential-oil-loaded lipid nanocarriers with anti-inflammatory activity between 2021 and 2026.

Author (Year)	EO/Nanocarrier	Major EO Constituent	Physicochemical Properties	Experimental Model/Route	Free EO as Control	Control Carrier	Reference Drug	Anti-Inflammatory Findings	Safety Assay	Bioassays Stages
Abdelhameed et al. (2023) [28]	*Eugenia supra-axillaris* EO/Nanoemulsion	D-Limonene	Size 56.9 ± 0.44 nm; ZP −1.84 mV; PDI NR; EE NR	Carrageenan-induced paw edema in rats; oral, 50 and 100 mg/kg	Yes	NR	Celecoxib and diclofenac	NE at 100 mg/kg produced 53.61% edema inhibition after 24 h; activity greater than free EO under the tested conditions	Acute oral toxicity; No mortality or toxic signs up to 2000 mg/kg	Preclinical in vivo
Mirković et al. (2025) [30]	*Pinus halepensis* EO/Liposomes	alpha-Pinene	Size 197.4 ± 3.81 nm; PDI 0.15 ± 0.002; ZP −35.61± 2.0 mV	Carrageenan-induced paw edema in rats; topical, 0.3 g	NR	NR	Hydrocortisone ointment 1%	Edema inhibition of 51.83% at 3 h (paw thickness 5.89 ± 0.26 mm) and 69.23% at 4 h (5.20 ± 0.08 mm); response of similar magnitude to hydrocortisone	No observable cutaneous irritation	Preclinical in vivo
Cristiano et al. (2022) [31]	Bergamot EO + ammonium glycyrrhizinate/Ultra-deformable lipid vesicles	NR	Size 129 ± 1 nm; PDI 0.120 ± 0.024; ZP −43.2 ± 1.6 mV; EE: AG 65.0 ± 2.0%; BEO 26.4 ± 1.3%	Methyl nicotinate-induced erythema in 10 healthy volunteers; topical	NR	NR	AG-only vesicles	BEO + AG vesicles significantly reduced erythema compared with AG-only vesicles (*p* < 0.001)	Topical tolerability in 10 healthy volunteers; no significant erythema variation versus saline	Exploratory clinical evidence
Hou et al. (2026) [32]	Garlic oil/PEG-stabilized lipid nanodisks	NR	Size 148 ± 3.0 nm; PDI 0.15 ± 0.02; ZP −0.2 ± 0.1 mV; EE 55.26 ± 0.04%	LPS-induced acute lung injury in male ICR mice; 50 mg/kg tail-vein injection	Yes	NR	NR	Reduced TNF-α, IL-6, lung wet/dry ratio and NO; increased IL-4 and IL-10; NF-kappaB inhibition and Keap1/Nrf2/HO-1/NQO1 activation	Not assessed	Disease-specific preclinical in vivo
Kotb et al. (2023) [34]	*Boswellia papyrifera* EO/SLN	n-Octyl acetate	Size 64.00 ± 5.66 nm; ZP −28.70 ± 0.42 mV; EE 77.24 ± 0.42%; sustained release for 8 h	UVB-induced skin photodamage in male Wistar rats; topical for 10 days	NR	NR	Vitamin A palmitate	Reduced IL-6 and NF-kappaB p65 and modulated MAPK and PI3K/AKT signaling	Skin histology/tolerability assessed	Disease/model-specific preclinical in vivo
Mohemmad et al. (2024) [35]	*Elettaria cardamomum* EO/NLC	NR	Size 150.13 ± 15.25 nm; PDI 0.449 ± 0.45; ZP −12.30 ± 1.9 mV; %EE of 91.95% ± 0.85%	Ethanol-induced gastric ulcer in rats; oral, 300 and 600 mg/kg	NR	NR	Omeprazole 20 mg/kg	Reduced IL-6 and TNF-α, increased IL-10, and reduced gastric ulcer area	NR	Disease-specific preclinical in vivo
Ahmed et al. (2024) [36]	*Elettaria cardamomum* EO/NLC	NR	NR	High-sugar/high-fat diet plus STZ-induced diabetes in male Wistar rats; oral, 28 days	NR	NR	Empagliflozin	Reduced fasting blood glucose and TNF-α/IL-6; findings were obtained in a disease-specific metabolic model	Four-week administration; liver, pancreas and kidney histology; no relevant alterations	Disease-specific preclinical in vivo
Shakeel et al. (2021) [37]	Clove EO/NLC	Eugenol	Size 120 ± 5.2 nm; PDI 0.148 ± 0.013; ZP −27.56 ± 1.9 mV; EE 84.53 ± 4.12%	FCA-induced arthritis in male Wistar rats; topical, 21 days	NR	NR	Diclofenac sodium gel 1%	Reduced paw edema and serum TNF-alpha, IL-1β, IL-6, and lysosomal enzymes	No observable skin irritation reported	Disease-specific preclinical in vivo
Gavzan et al. (2025) [39]	*Pelargonium graveolens* EO/Nanoemulsion	Citronellol	Size 553.1 ± 3.1 nm; PDI 0.113 ± 0.007; ZP +28.4 ± 1.2 mV (SD NR)	Carrageenan- and formalin-induced paw edema in mice; 50 and 100 mg/kg	Yes	NR	NR	NE inhibited edema in carrageenan and formalin models; free EO at 10 mg/kg was inactive under the tested conditions	Non-hemolytic assessment before in vivo testing	Preclinical in vivo
Gava et al. (2025) [40]	*Ocimum gratissimum* EO/Nanoemulsion	(E)-beta-Ocimene	Size 112 ± 0.9 nm; PDI 0.20 ± 0.00; ZP −5.2 ± 0.1 mV at D0/4 °C; stability up to 60 days at 4 °C	LPS-stimulated RAW 264.7 macrophages; EO 2.5 and 5 µg/mL	Yes	NR	NR	NE inhibited TNF-α, IL-6 and IL-1, whereas free EO suppressed only TNF-α; ROS was also reduced	HET-CAM/CAM-TBS, NRU-3T3 phototoxicity and cytotoxicity in L929, RAW 264.7 and HaCaT cells	In vitro mechanistic evidence
Koushik et al. (2025) [41]	*Melissa officinalis* EO/Nanoemulsion gel	NR	Droplet size 127.31 ± 2.84 nm; PDI 0.177; ZP −25.0 ± 1.5 mV; EE 95.63% ± 0.47%	Carrageenan-induced paw edema in Wistar rats; topical	NR	NR	Diclofenac gel 2.5% *w*/*w*	At 30 min, nanoemulgel reduced paw edema volume to 0.25 mL versus 0.32 mL for diclofenac; conditions were not sufficient to establish equivalence	Acute dermal irritation (OECD 404); slight erythema at 1 h, resolved by 24 h; no edema	Preclinical in vivo
Mohammadifar et al. (2021) [42]	*Mentha piperita* + *Rosmarinus officinalis* EOs/Nanoemulsion gel	NR	NR	MIA-induced knee osteoarthritis in male rats; topical, 14 days	NR	NR	Diclofenac sodium gel 1%	Increased paw-withdrawal threshold, reduced withdrawal frequency (*p* < 0.01), and improved synovial histology; SOD/GPx increased and MDA decreased	Dermal irritation and acute dermal toxicity assessments reported	Disease-specific preclinical in vivo
Yetukuri & Umashankar (2025) [43]	*Kunzea ericoides* EO/Nanoemulsion gel	Isolimonene	Size 112.38 ± 1.45 nm; PDI 0.203 ± 0.012; ZP −29.0 ± 1.1 mV	Carrageenan-induced paw edema in rats; topical	NR	NR	Dicloran^®^ gel 2.5%	Significant reduction in edema (*p* = 0.005 at 60 min); response compared with diclofenac reference	Acute dermal irritation; formulation classified as non-irritant	Preclinical in vivo
Esmaeili et al. (2022) [44]	Cinnamon and clove EOs/Nanoemulsion-based nanogels	Cinnamaldehyde (cinnamon); Eugenol (clove)	Cinnamon NE 28 ± 6 nm; clove NE 12 ± 3 nm	Carrageenan paw edema, formalin and hot-plate tests in male Wistar rats; topical	NR	NR	NR	Significant anti-inflammatory and antinociceptive activity; cinnamon formulation showed greater activity in the chronic formalin phase	Tolerability inferred from absence of behavioral distress	Preclinical in vivo
Dakhlaoui et al. (2024) [45]	*Eucalyptus cladocalyx* EO/Nanoemulsion	o-Cymene	Size 44.9 ± 0.12 nm; PDI 0.49 ± 0.05; stability 4 months	LPS-stimulated RAW 264.7 macrophages	Yes	NR	NR	Both NE and free EO inhibited NO, IL-6, and TNF-α; superiority of nanoencapsulation was not established	NR	In vitro
Shafiq et al. (2025) [46]	Clove EO/NE-based topical cream	Eugenol	Size: ~190 nm; PDI: 0.08; EE: 94.54%	Inflammatory dorsal skin wound model in male albino mice	NR	NR	Dicloran gel	Cream reduced inflammation and promoted wound healing	NR	Preclinical in vivo

Abbreviations: EO, essential oil; NE, nanoemulsion; NLC, nanostructured lipid carrier; SLN, solid lipid nanoparticle; PDI, polydispersity index; ZP, zeta potential; %EE, encapsulation efficiency; NR, not reported; LPS, lipopolysaccharide; STZ, streptozotocin; MIA, monosodium iodoacetate; FCA, Freund’s complete adjuvant; AG, ammonium glycyrrhizinate; BEO, bergamot essential oil.

## 8. Discussion

The reviewed works indicated that EO-loaded lipid nanocarriers can show anti-inflammatory activity in specific experimental settings. It has already been demonstrated that EO interact with the lipid matrices, acting also as a structural excipient in the formation of nanoparticles, driven by hydrophobic interactions. It is worth mentioning that the biological responses varied according to the EO, nanocarrier composition, experimental model, route of administration and efficacy assay models tested. Direct comparisons with the corresponding free EO and control formulations were available only in a few reports. Therefore, the contribution of nanoencapsulation cannot be distinguished from the effects of the EO itself, lipids, surfactants, or other excipients in all cases.

Anti-inflammatory responses were also formulation-specific and varied according to EO composition, carrier composition, dose, route of administration, experimental model and endpoint. The lipid matrix biodegradability rate modulates the in vitro release profile and the biological performance of the nanocarriers [47]. Due to differences in physicochemical properties and affinity for the carrier matrix, different constituents of the same EO can exhibit distinct release kinetics. Accordingly, direct comparisons among nanocarriers cannot be assumed, as there are no available data under equivalent experimental conditions [48]. Similar responses in magnitude to those of a reference drug in selected models do not demonstrate therapeutic equivalence.

Some limitations affect the translational consolidation of essential-oil-based nanocarriers, such as the phytochemical standardization of the bioactive compounds. The absence of harmonized criteria for reporting chemotype, including the systematic reporting of voucher specimen, gas chromatography–mass spectrometry (GC–MS) profiles and identification of constituents, represents the main challenge to the reproducibility, comparison among nanoformulations and approval [49]. This gap was also evident in the present review, in which a substantial number of works did not specify the chemotype of the encapsulated oil nor the analytical method used to quantify the active. EO are chemically heterogeneous mixtures whose composition depends on botanical origin, chemotype, plant organ, harvest season, geographic location, extraction method and storage conditions [9].

Another challenge concerns the manufacturing process and the reproducibility of nanostructured formulations at the large scale. The encapsulation of bioactive compounds is particularly sensitive to batch-to-batch variability. Small differences in lipid composition, surfactant concentration or process parameters, as homogenization pressure, sonication amplitude and temperature, can change particle size distribution, polydispersity index and encapsulation efficiency. It was also noted that several promising nanoproducts have not been translated to industrial scale because some industrial techniques require careful re-optimization to work with chemically complex natural mixtures [24]. The systematic implementation of quality-by-design (QbD) strategies, supported by design-of-experiments (DoE) tools, represents the most consistent route to bridge the laboratory–industry gap, allowing the development of essential-oil-loaded nanocarriers in a robust process [47].

Moreover, there are persistent gaps in the regulation of nano-specific products guidance among the U.S. Food and Drug Administration (FDA), European Medicines Agency (EMA) and Brazilian Health Regulatory Agency (ANVISA), as well as the limited adaptation of conventional International Council for Harmonisation (ICH) guidelines Q8 on pharmaceutical development, Q9 on quality risk management and Q10 on the pharmaceutical quality system to the specificities of nanoscale products. Specifically, the approval of lipid nanocarriers loaded by EO crosses the interface between conventional drug development and botanical-product regulation. For botanical-active requirements, there are some challenges in the identification of the source plant, chemotype and the elucidation of the GC-MS profile [49]. Until a specific regulatory framework for herbal-based nanodrugs becomes available, the best strategy is the alignment of both sets of analyses, providing robust information about the new systems developed.

Several formulations composed of different anti-inflammatory bioactive exhibited suitable physicochemical properties. However, the inflammatory microenvironment has to be considered in such analyses. Local changes in pH, enzymatic activity and oxidative conditions can influence nanocarrier stability, EO uptake and in vitro release profiles. Such inflammation-related conditions were not systematically evaluated across the works included in this review. Therefore, the physicochemical stability and in vitro release profiles observed under conventional experimental conditions should not necessarily reflect the behavior of the formulations at inflamed tissues. Future works should evaluate nanocarriers under the inflammatory microenvironment.

On the other hand, regarding safety, the assays mainly included cytotoxicity, local tolerability and dermal irritation. Favorable results from these assays are relevant to the specifically evaluated formulation. In addition, short-term or acute assessments do not provide sufficient evidence regarding repeated-dose or long-term safety. Therefore, the safety profile of each formulation should be interpreted according to the type and duration of the toxicological assessment performed.

In the efficacy assays, acute paw edema, chronic osteoarthritis and LPS-stimulated macrophages were the most commonly used efficacy models, in which the lipid-based formulations have shown significant anti-inflammatory effects in these models. In general, different levels of suppression of were observed for TNF-α, IL-6, IL-17 and NO via NF-κB and GSK-3β as experimental evidence. These findings supported the anti-inflammatory activity under the specific conditions. In addition, the multicomponent nature of EO has to be considered when interpreting these biological effects. Although this multitarget activity should contribute to the overall anti-inflammatory response, it also makes it difficult to attribute to a specific constituent or molecular target. Furthermore, potential nonspecific or off-target effects remain insufficiently characterized and require further investigation [50]. Still, the preclinical evidence should also be interpreted cautiously, since information on sample-size justification, randomization and blinding, that intended to reduce experimental bias, are not uniformly available. Further works should integrate mechanistic readouts (NF-κB, COX-2, iNOS, MAPK and Keap1/Nrf2/HO-1 pathway analysis) besides those purely symptomatic endpoints. Clinical assays remain restricted. There are only a few clinical studies available, predominantly focused on dermatological, oral or veterinary applications.

Moreover, the contribution of the EO nanoencapsulation to the anti-inflammatory responses should be considered together with the related experimental controls. As shown here, comparisons with the corresponding free EO and control nanocarriers were not consistently performed. Therefore, the potential contribution of lipids, surfactants or other excipients to the biological response cannot be completely discarded. Furthermore, comparing the efficacy of the nanoformulations with that of conventional anti-inflammatories also requires attention to avoid misinterpretations. Similar responses observed under a particular experimental model indicate that the nanoformulation and reference drug produced effects of similar magnitude under the tested conditions, but they did not demonstrate pharmacological or therapeutic equivalence. Further analyses will be essential to ensure such similarity. Although the works summarized in this review provided predominantly preclinical evidence, human investigations remain limited. The available clinical data were based on exploratory topical evaluation rather than controlled efficacy trials. Therefore, the current evidence cannot establish clinical efficacy of EO-loaded lipid nanocarriers.

Future biological investigation of anti-inflammatory nanosystems can be improved by combining conventional inflammatory outcomes with mechanistic endpoints. Analyses should include NF-κB, COX-2, iNOS, MAPK, and Keap1/Nrf2/HO-1 pathways, together with cytokine measurements and histological or functional outcomes. This will provide a more comprehensive understanding of the mechanisms associated with the observed anti-inflammatory responses.

## 9. Conclusions

The present literature review investigated works regarding essential oil-loaded lipid nanoparticles for anti-inflammatory applications published in the period between 2021–2026. The available reports suggested that the lipid nanocarriers contributed to overcome the intrinsic limitations of free EO, including volatility, oxidative or photolytic instability and low aqueous dispersibility. However, the extent of this benefit is formulation-specific.

In tested acute, subacute and chronic experimental models, some nanoformulations produced anti-inflammatory responses of similar magnitude to hydrocortisone, diclofenac or other reference drugs. Since free EO, control carriers, dosage, routes of administration and experimental conditions were not consistently equivalent, these are considered preliminary findings that cannot be assumed as therapeutically equivalent to conventional drugs. These works varied regarding EO composition, carrier system, physicochemical properties, route of administration, dosage, treatment period, experimental model and biological endpoints. Most of the biological evidence remains preclinical and includes mechanistic in vitro investigations, acute animal experiments and disease-specific animal models. Evidence in humans is scarce and is based on exploratory evaluation rather than controlled efficacy trials. Safety findings are formulation- and assay-dependent, focused on cytotoxicity, local tolerance and skin-irritation assessments. These data do not determine repeated-dose or long-term safety.

The consolidation of EO-loaded lipid nanocarriers as anti-inflammatory candidates still depends on phytochemical standardization, comparative controls, reproducibility, mechanistic preclinical evaluation and an appropriate regulatory framework. The integration of standardized phytochemical control, quality-by-design manufacturing, mechanistic preclinical evaluation and proactive regulatory alignment is still necessary.

## Figures and Tables

**Table 1:** Main non-steroidal anti-inflammatory drugs used in clinical practice: COX selectivity, oral dosing, therapeutic indications, and adverse effects.

COX Selectivity	Drug	Clinical Oral Dose	Main Mechanism/Prescription	Side Effects
Non-selective, irreversible COX-1/COX-2 inhibitor (salicylate)	Acetylsalicylic acid	325–650 mg every 4–6 h for pain/fever (max 4 g/day) ^1^; 75–100 mg once daily for antiplatelet prophylaxis	Irreversible acetylation of platelet COX-1; antiplatelet effect; analgesic, antipyretic, anti-inflammatory; secondary cardiovascular prevention	GI ulceration/bleeding; bronchospasm in aspirin-sensitive asthma; salicylism (tinnitus) at high doses; Reye’s syndrome in children/adolescents with viral illness ^2^
Non-selective COX inhibitor (propionic acid derivative)	Ibuprofen	200–400 mg every 4–6 h for mild–moderate pain; max 1200 mg/day OTC, up to 3200 mg/day prescription ^3^	Reversible COX-1/COX-2 inhibition; analgesic, antipyretic, anti-inflammatory; osteoarthritis (OA), rheumatoid arthritis (RA), dysmenorrhea	GI dyspepsia, ulcer/bleeding; renal impairment; modest dose-related cardiovascular (CV) thrombotic risk; antagonism of aspirin antiplatelet effect
Non-selective COX inhibitor (propionic acid derivative)	Naproxen	250–500 mg every 12 h (max 1000–1250 mg/day; up to 1500 mg/day for limited periods)	Reversible COX-1/COX-2 inhibition; OA, RA, ankylosing spondylitis (AS), acute gout, dysmenorrhea	GI ulceration/bleeding (high among non-selective NSAIDs); renal effects; comparatively neutral CV profile in meta-analyses
Preferential COX-2 inhibitor (acetic acid derivative)	Diclofenac	50 mg two or three times daily, or 75 mg twice daily; max 150 mg/day (OA), 200 mg/day (RA)	Reversible COX-2-leaning inhibition; OA, RA, AS, acute pain, dysmenorrhea	GI ulceration/bleeding; hepatotoxicity (transaminase elevation); CV thrombotic risk comparable to coxibs at high dose; renal toxicity
Non-selective COX inhibitor (acetic acid derivative, indole)	Indomethacin	25–50 mg two to three times daily; max 150–200 mg/day	Potent COX-1/COX-2 inhibition; moderate-to-severe RA, OA, AS, acute gout, bursitis/tendinitis	High GI toxicity; CNS effects (headache, dizziness, confusion); renal impairment; hematological toxicity; on the Beers list for older adults
Non-selective COX inhibitor (acetic acid derivative, pyrrolizidine)	Ketorolac	20 mg single oral dose, then 10 mg every 4–6 h; max 40 mg/day; combined parenteral + oral use ≤5 days ^4^	Strong reversible COX-1/COX-2 inhibition; short-term management of moderate-to-severe acute pain (opioid-sparing)	Boxed warnings for GI bleeding, renal failure, bleeding diathesis; CV thrombotic risk; contraindicated in CABG, advanced renal disease, active peptic ulcer
Preferential COX-2 inhibitor (oxicam)	Meloxicam	7.5 mg once daily; may be increased to 15 mg once daily (max)	COX-2-preferential reversible inhibition; OA, RA, juvenile idiopathic arthritis	GI events less frequent than with non-selective NSAIDs at 7.5 mg/day, but COX-2 selectivity is dose-dependent and partially lost at 15 mg/day ^5^; renal and CV class warnings apply
Selective COX-2 inhibitor (sulfonamide-coxib)	Celecoxib	OA: 200 mg once daily or 100 mg twice daily; RA: 100–200 mg twice daily; AS: 200–400 mg/day; max 400 mg/day	Selective reversible COX-2 inhibition; OA, RA, AS, acute pain, primary dysmenorrhea, familial adenomatous polyposis	Lower GI toxicity than non-selective NSAIDs; CV thrombotic risk non-inferior to ibuprofen/naproxen at moderate dose (PRECISION); contraindicated in sulfonamide allergy and CABG perioperative use
Selective COX-2 inhibitor (methylsulfone-coxib; EMA-approved, not FDA-approved) ^6^	Etoricoxib	OA: 30–60 mg once daily; RA/AS: 60–90 mg once daily; acute gout: 120 mg once daily for ≤8 days	Highly selective reversible COX-2 inhibition; OA, RA, AS, acute gouty arthritis, acute postoperative dental pain (EMA-licensed indications)	Dose-dependent hypertension and oedema; CV thrombotic risk (basis of FDA non-approval, 2007); contraindicated in uncontrolled hypertension, ischemic heart disease, cerebrovascular disease and severe heart failure; hepatotoxicity

^1^ For chronic anti-inflammatory use of aspirin (e.g., rheumatic disease), prescribing references describe initial doses up to 3 g/day in divided doses, individualized and monitored against plasma salicylate (target 150–300 µg/mL); analgesic OTC ceiling is 4 g/day. ^2^ Aspirin is contraindicated in children and adolescents (<19 years) with viral illness owing to the epidemiological association with Reye’s syndrome. ^3^ The FDA-approved OTC ceiling is 1200 mg/day; the prescription ceiling stated in current Motrin^®^ (ibuprofen) labelling is 3200 mg/day. ^4^ The combined duration of parenteral plus oral ketorolac administration in adults must not exceed 5 days according to FDA warning, owing to dose- and duration-dependent risk of GI bleeding, acute kidney injury and bleeding events. ^5^ Meloxicam is regarded by the FDA as a non-selective NSAID for labelling purposes; pharmacological reviews more frequently classify it as COX-2-preferential at 7.5 mg/day, with progressive loss of selectivity at 15 mg/day. ^6^ Etoricoxib is approved by the EMA and in more than 60 countries but was not approved by the FDA; the FDA Arthritis Advisory Committee voted 20 to 1 against approval in April 2007 because of concerns regarding cardiovascular thrombotic and hypertensive risk.

## Data Availability

No new data were created or analyzed in this study. Data sharing is not applicable to this article.

## Abbreviations

AG	Ammonium glycyrrhizinate
AKT	Protein kinase B
ANVISA	Brazilian Health Regulatory Agency
AP-1	Activator protein 1
AS	Ankylosing spondylitis
BEO	*Bergamot essential* oil
CABG	Coronary artery bypass grafting
CAM-TBS	Chorioallantoic membrane–trypan blue staining
CAS	Chemical Abstracts Service
CAT	Catalase
CNS	Central nervous system
COX	Cyclooxygenase
COX-1	Cyclooxygenase-1
COX-2	Cyclooxygenase-2
CV	Cardiovascular
DoE	Design of experiments
DSPC	1,2-Distearoyl-sn-glycero-3-phosphocholine
DSPE-mPEG2000	1,2-Distearoyl-sn-glycero-3-phosphoethanolamine-N-[methoxy(polyethylene glycol)-2000]
EE	Encapsulation efficiency
ELISA	Enzyme-linked immunosorbent assay
EMA	European Medicines Agency
EO	Essential oil
ERK	Extracellular signal-regulated kinase
FCA	Freund’s complete adjuvant
FDA	U.S. Food and Drug Administration
GC-MS	Gas chromatography-mass spectrometry
GI	Gastrointestinal
GMO	Genetically modified organism
GPx	Glutathione peroxidase
GSK-3β	Glycogen synthase kinase 3 beta
HEPES	4-(2-Hydroxyethyl)-1-piperazineethanesulfonic acid
HET-CAM	Hen’s egg test-chorioallantoic membrane
HO-1	Heme oxygenase-1
ICH	International Council for Harmonisation
ICR	Institute of Cancer Research
IL-1	Interleukin-1
IL-1β	Interleukin-1 beta
IL-4	Interleukin-4
IL-6	Interleukin-6
IL-10	Interleukin-10
IL-17	Interleukin-17
iNOS	Inducible nitric oxide synthase
JNK	c-Jun N-terminal kinase
Keap1	Kelch-like ECH-associated protein 1
LD50	Median lethal dose
LPS	Lipopolysaccharide
MAPK	Mitogen-activated protein kinase
MDA	Malondialdehyde
MEDLINE	Medical Literature Analysis and Retrieval System Online
MEP	Methylerythritol phosphate
MIA	Monosodium iodoacetate
MIC	Minimum inhibitory concentration
MVA	Mevalonate
NE	Nanoemulsion
NF-κB	Nuclear factor kappa B
NLC	Nanostructured lipid carrier
NO	Nitric oxide
NQO1	NAD(P)H quinone dehydrogenase 1
NR	Not reported or not confirmed in the currently extracted study information
Nrf2	Nuclear factor erythroid 2-related factor 2
NRU-3T3	3T3 neutral red uptake assay
NSAID	Non-steroidal anti-inflammatory drug
OA	Osteoarthritis
OTC	Over-the-counter
PDI	Polydispersity index
PEG	Polyethylene glycol
PGE2	Prostaglandin E2
PI3K	Phosphoinositide 3-kinase
qPCR	Quantitative polymerase chain reaction
QbD	Quality by design
RA	Rheumatoid arthritis
RH	Relative humidity
ROS	Reactive oxygen species
SLN	Solid lipid nanoparticle
SOD	Superoxide dismutase
STZ	Streptozotocin
TGF-β1	Transforming growth factor beta 1
TGP	Total glucosides of peony
TNF-α	Tumor necrosis factor alpha
UVB	Ultraviolet B
ZP	Zeta potential
